# Super-Resolution Reconstruction and Detector Geometric Error Correction for Parallel-Beam Low-Resolution Multi-Detector SPECT: A Proof of Concept

**DOI:** 10.3390/tomography12020023

**Published:** 2026-02-12

**Authors:** Zhibiao Cheng, Jun Zhang, Ping Chen, Junhai Wen

**Affiliations:** 1 State Key Laboratory of Extreme Environment Optoelectronic Dynamic Measurement Technology and Instrument, North University of China, Taiyuan 030051, China; 2 Shanxi Key Laboratory of Intelligent Detection Technology and Equipment, North University of China, Taiyuan 030051, China; 3 School of Information and Communication Engineering, North University of China, Taiyuan 030051, China; 4 Department of Biomedical Engineering, School of Life Science, Beijing Institute of Technology, Beijing 100081, China; 5 College of Computer Science and Technology, Taiyuan University of Technology, Taiyuan 030600, China

**Keywords:** SPECT, parallel-beam, multi-detector, super-resolution reconstruction, geometric error correction

## Abstract

**Objectives:** Due to collimator limitations, Single-Photon Emission Computed Tomography (SPECT) suffers from relatively low spatial resolution, which hampers the detection of small lesions. This study proposes a super-resolution (SR) reconstruction algorithm for a parallel-beam, low-resolution (LR) multi-detector SPECT system and employs a neural network to estimate and correct for geometric errors in the LR detectors. **Methods:** A parallel-beam LR multi-detector SPECT system is presented, in which the detectors perform relative sub-pixel shifts. At each sampling angle, an SR reconstruction algorithm synthesizes high-resolution (HR) SPECT images from LR projections acquired by four offset LR detectors. To correct for geometric errors among these detectors, a randomly distributed gamma point source was designed to generate training data. A neural network was then employed to estimate the geometric errors, thereby refining the SR reconstruction. **Results:** Numerical simulation demonstrated that the proposed neural network could accurately identify the displacement-based geometric errors of the LR detectors. Utilizing these estimated parameters to correct the SR reconstruction process yielded results comparable to those obtained from direct reconstruction of HR projections, achieving a two-fold resolution improvement. **Conclusions:** Preliminary proof-of-principle for SR reconstruction in a parallel-beam LR multi-detector SPECT system was established. Further validation of the hardware performance is warranted.

## Simple Summary

Single-Photon Emission Computed Tomography (SPECT) is characterized by inherently low spatial resolution, which limits its ability to detect small lesions. To address this, we propose a novel super-resolution (SR) reconstruction method for a multi-detector, low-resolution (LR) SPECT system. Our approach integrates data from offset detectors and employs a neural network to correct for inter-detector geometric errors. Numerical simulations verify that this method effectively doubles the image resolution, presenting a promising solution for advancing SPECT imaging.

## 1. Introduction

Single Photon Emission Computed Tomography (SPECT) is a molecular imaging technique based on detecting radioactive tracers. It visualizes functional changes—such as cellular metabolism, molecular binding, and signal transmission—in living tissues and organs at the molecular level, thereby detecting early functional abnormalities and pathology associated with diseases [1,2,3]. Characterized by a larger field of view (FOV), parallel-beam SPECT holds a significant market share and sees wide application. Its sensitivity remains constant across distances and can produce images identical in size and orientation to the target. However, due to the physical constraints of collimator hole length, aperture size, and septal thickness, the sensitivity and spatial resolution of SPECT cannot be improved simultaneously. Its relatively low spatial resolution thus limits the visualization and detection of small lesions [4].

The pursuit of higher-resolution SPECT imaging has spurred extensive research into reconstruction algorithms [5]. Iterative algorithms, particularly Maximum Likelihood Expectation Maximization (MLEM), are widely adopted for their robust mathematical foundation, which provides a principled framework for modeling emission and detection processes [6]. To balance image recovery with noise suppression, various regularization techniques—including total variation (TV) and its derivatives, as well as non-local regularization—have been applied within the reconstruction pipeline [7,8]. Anatomical priors have also been leveraged to enhance image sharpness, giving rise to methods such as the Gibbs prior, mutual information, and kernelized expectation maximization (KEM) [9,10,11]. Recently, deep learning (DL) has further diversified the field. DL-based approaches can be broadly categorized into three groups: DL-aided projection domain processing [12], image-domain enhancement [13,14,15,16], and hybrid schemes that integrate DL with classical iterative reconstruction [17,18,19,20]. Furthermore, the fusion of multi-modal data has shown promise in generating clearer images with richer diagnostic information [21].

Improving imaging resolution can also be pursued through hardware refinements, with the primary challenge lying in advancing detector technology—specifically its scintillation crystal, photomultiplier tube, and collimator. However, the pursuit of superior hardware is not only costly but also constrained by inherent performance limits. An alternative approach involves multi-image super-resolution (SR) for SPECT—which often relies on detector or subject movement to achieve higher spatial resolution [22,23,24,25,26]. By introducing richer priors, this method enhances reconstruction fidelity. Its effectiveness, however, depends on precise calibration. Since motion and registration inaccuracies may cause systematic errors, accurate correction of geometric effects is essential during reconstruction [5].

In this study, we extend our previously proposed two-dimensional SR SPECT acquisition and reconstruction methodology into three dimensions and derive the corresponding SR reconstruction algorithm [24]. To avoid additional sampling time and prevent errors induced by the motion of low-resolution (LR) detectors, we designed a configuration comprising four uniformly distributed LR detectors within the rotational space of the detector assembly. These four detectors exhibit controlled positional offsets relative to their central axes, with displacements in the transverse and axial directions typically set at the scale of high-resolution (HR) pixel size. This arrangement effectively simulates shifting a single LR detector within its plane at each sampling angle, thereby acquiring four slightly variant LR images from the same viewing perspective. Due to inherent manufacturing and mechanical precision limitations, the actual detector positions may exhibit slight discrepancies from their intended coordinates. Neglecting these geometric misalignments during SR reconstruction would significantly degrade the accuracy of the resultant SR projection images. Once assembled, the geometric errors induced by detector displacement in our multi-detector SPECT system remain constant. To address this, we developed a geometric error correction method. The approach involves pre-imaging an arbitrary gamma point source model and applying a neural network-based algorithm to quantify the true displacement deviations between the HR and LR projections. These quantified deviations are then integrated into our SR reconstruction framework, enabling super-resolved reconstruction for the parallel-beam LR multi-detector SPECT system.

The novelty of this study lies in proposing a novel LR multi-detector SPECT SR imaging method with relative displacement offsets and designing a neural network to correct these offsets. The main contribution of this study is to provide a new perspective for multi-detector SR SPECT imaging.

The remainder of this paper is organized as outlined below. Section 2 describes the parallel-beam LR multi-detector SPECT concept, the SR reconstruction method, and the detector geometric error correction approach. Section 3 presents the dataset and training details, quantitative assessment metrics, and the experimental results. Section 4 discusses the validity and limitations of these methods. Finally, Section 5 concludes the paper.

## 2. Materials and Methods

### 2.1. Description of the Parallel-Beam LR Multi-Detector SPECT

**Figure 1:**
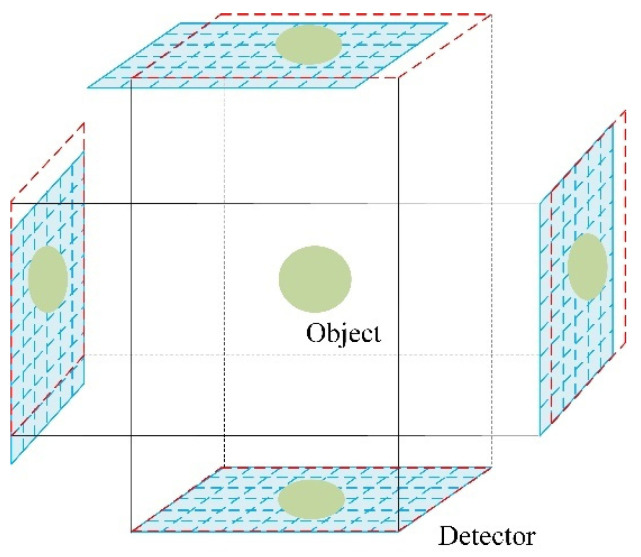
Parallel-beam LR multi-detector SPECT model.

Employing multi-image SR techniques, a parallel-beam LR multi-detector SPECT is proposed to enhance spatial resolution. SPECT component includes four independent parallel-beam detectors. As illustrated in Figure 1, the four red dashed boxes represent uniformly distributed reference detector positions around the target. In contrast, at each reference position, our detectors are configured to perform sub-pixel-level relative motion. This enables the acquisition of multiple slightly varied LR projection images from the same angular view, thereby complementing spatial information across images. The blue dashed lines in Figure 1 depict the configuration of our multi-detector setup.

**Figure 2:**
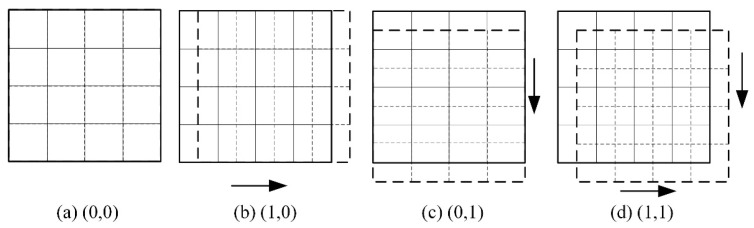
Offsets of the LR detectors relative to their reference positions.

For ease of description, the image in parallel-beam SPECT imaging is represented in terms of pixel values. Let the LR and HR projection images have sizes of *S*_*l*_ × *S*_*l*_ and *S_h_* × *S*_*h*_ pixels, respectively. The magnification factor is then given by: R = *S*_*h*_/*S*_*l*_. At each sampling angle *φ* during the rotation of the LR detector around the center of the object, the detector is translated within the detector plane by integer multiples of the corresponding HR pixel size in different directions. At least *R*^2^ LR projection images are acquired at different positions [26]. Generally, greater sub-pixel displacements between LR images provide richer non-redundant information for SR reconstruction. The relative displacement of the LR detectors is set to equal the width of a single pixel in the corresponding HR image. This study adopts an upscaling factor of *R* = 2 as an example. At each sampling angle *φ*, the displacement of each LR detector relative to its initial position is set to (0, 0), (1, 0), (0, 1), and (1, 1), as shown in Figure 2. The solid lines represent the initial positions of the LR detectors, while the dashed lines depict their positions after applying the respective sub-pixel translations relative to the reference.

By rotating the multi-LR-detector assembly around the target, four LR projections of the same scene are obtained, which can be considered as being derived from a corresponding HR projection at the identical sampling angle. This is achieved by shifting the HR projection image within the detector plane by a predefined displacement in the corresponding direction, followed by a downsampling degradation process. Assuming *h* is the HR projection at an arbitrary sampling angle, the *r*-th LR projection *l*_*r*_ can be modeled as:

(1) $$l_{r}={\mathrm{DH}}_{r}T_{r}h=W_{r}h+n$$

In this case, *W*_*r*_ represents the operations of downsampling (*D*), blurring (*H*_*r*_), and motion deformation (*T*_*r*_). *n* denotes noise.

**Figure 3:**
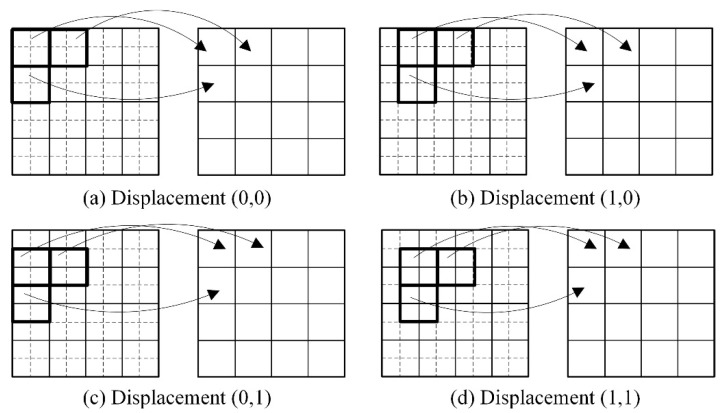
Schematic illustration of the relationship between the HR projection image and the ideal LR projection images under different displacements.

Figure 3 visually illustrates the relative positional relationship between HR and LR projection images under ideal conditions. The value of a single pixel in the LR projection image is equal to the sum of the values of the HR pixels within the corresponding region, denoted as block *S_R_*, in the HR projection image. This region is highlighted by the bold solid-line box in the figure. Specifically, it can be described as:

(2) $$P_{L}(x,y)=\sum_{\left(x^{\prime },y^{\prime }\right)\in S_{R}}P_{H}^{\prime }(x^{\prime },y^{\prime })$$

here, $P_{L}(x,y)$ represents the pixel value at position (x,y) in the LR projection image, while $P_{H}^{\prime }(x^{\prime },y^{\prime })$ denotes the pixel value at position $\left(x^{\prime },y^{\prime }\right)$ in the HR projection image. The summation $\left(x^{\prime },y^{\prime }\right)$ is performed over the corresponding *R* × *R* region block $S_{R}$. The resultant grayscale value in the LR image is governed by the blurring introduced by the point spread function at the relevant location.

### 2.2. Proposed SR Reconstruction Algorithm

A new SR reconstruction algorithm has been developed for a parallel-beam LR multi-detector SPECT system. This system acquires four LR projections, each with unique sub-pixel offsets, under the same viewing angle. This data provides the complementary information required for multi-frame SR techniques. In the first phase, the algorithm generates a single SR projection image for every sampling angle. Standard tomographic reconstruction methods are then applied to this set of SR projections to obtain the final HR reconstructed volume.

At each sampling angle *φ*, a multi-image SR algorithm is used to generate a single SR projection image from the *R*^2^ LR projection images. The HR projection image is denoted as $p_{H\varphi }$, and the *r*-th LR projection image is denoted as ${\vec{p}}_{L\varphi r}$. The SR reconstruction algorithm proceeds through the following steps:

(a) Generate an initial HR projection image $p_{H\varphi }$ (that is, $p_{H\varphi (k=0)}$).
(b) The estimated HR projection image $p_{H\varphi rk}$ at the *r*-th translation position obtained at the *k*-th iteration, based on the degradation relationship $W_{r}$, generates the estimated LR projection image at the *r*-th movement position of the LR detector, denoted as:
  (3) $$p_{L\varphi rk}=W_{r}p_{H\varphi rk}$$
(c) To generate a new HR projection image at the *r*-th movement position, the adjustment weight $\Delta p_{H\varphi rk}$ can be represented as follows:
  (4) $$\Delta p_{H\varphi rk}=W_{r}^{T}({\vec{p}}_{L\varphi r}-p_{L\varphi rk})\times \lambda$$
  where *λ* is the adjustment step size.
(d) Generate a new estimated HR projection image $p_{H\varphi (r+1)k}$ at the (*r* + 1)-th movement position.
  (5) $$p_{H\varphi (r+1)k}=p_{H\varphi rk}+\Delta p_{H\varphi rk}$$
(e) Repeat steps (b~d). If all *R*^2^ adjustments are completed, proceed to the (*k* + 1)-th iteration, repeating the new *R*^2^ adjustments, until the iteration stopping condition is met: reaching the maximum number of iterations or $||{\vec{p}}_{L\varphi r}-p_{L\varphi rk}||<threshold$ (the maximum number of iterations and *threshold* are usually empirical values). The SR projection image at the sampling angle *φ* will be obtained.
(f) Repeat steps (a~e) to obtain the SR projection images for all sampling angles.
(g) Use MLEM reconstruction method to further reconstruct the SR SPECT image [27].

At the same sampling angle *φ*, a linear interpolation method is employed to align the estimated HR projection images obtained at each position into the same coordinate system. The coordinate system of the initial LR projection image is set as the reference coordinate system. The initial estimate of the HR projection image is obtained by upsampling the observed *R*^2^ LR projections, summing all the upsampled images, and averaging them. This helps achieve faster iterative convergence. The termination condition of the iteration is described as follows: the iteration is terminated when the Euclidean norm of the difference between the actual LR projections ${\vec{p}}_{L\varphi r}$ and the estimated LR projections $p_{L\varphi rk}$ falls below a predefined threshold. For practical implementation across different system configurations, the maximum number of iterations and the termination threshold are typically determined empirically, representing a trade-off between image quality and computational cost. The pseudocode of our SR algorithm is summarized in Algorithm 1.
**Algorithm 1.** SR SPECT reconstruction1: At each sampling angle *φ* as the LR detector rotates around the object, *R*^2^ LR projections $\left\{{\vec{p}}_{L\varphi r}\right\}$ are obtained by our projection acquisition method. 2: **for** *φ* = 1 … all angles 3:     The initial estimated SR projection $p_{H\varphi (k=0)}$ is generated at each sampling angle *φ*. 4:     **loop** k = 1 … maximum iteration number 5:       **for** m = 1 …*R*^2^ 6:           The *r*-th estimated LR projection $p_{L\varphi rk}=W_{r}p_{H\varphi rk}$ at each sampling angle φ is computed by Formula (1). 7:          The new estimate of $p_{H\varphi (r+1)k}$ is computed by Formula (5). 8:       **end** 9:       **if** $||{\vec{p}}_{L\varphi r}-p_{L\varphi rk}|{|}_{2}<\;\mathrm{threshold}$ or the maximum iteration number is reached then 10:      terminate 11:      loop 12:      **end** 13:   **end** 14: **end** 15: The SR image is reconstructed from the SR projection via MLEM reconstruction algorithm.

### 2.3. Proposed Detector Geometric Error Correction Algorithm

Ideally, the offset of the LR detector should follow the pattern shown in Figure 2. In practice, however, the displacement accuracy is constrained by the physical limits of the system hardware, and the positions for acquiring multiple LR projections are fixed. Consequently, the offset of the LR detector relative to its initial position is often not an exact integer multiple of the pixel size, typically deviating from the theoretical value by within ±1 pixel. To address this, a neural network algorithm was proposed to characterize the misalignments of the LR detectors, enabling their subsequent correction within the SR reconstruction framework.

**Figure 4:**
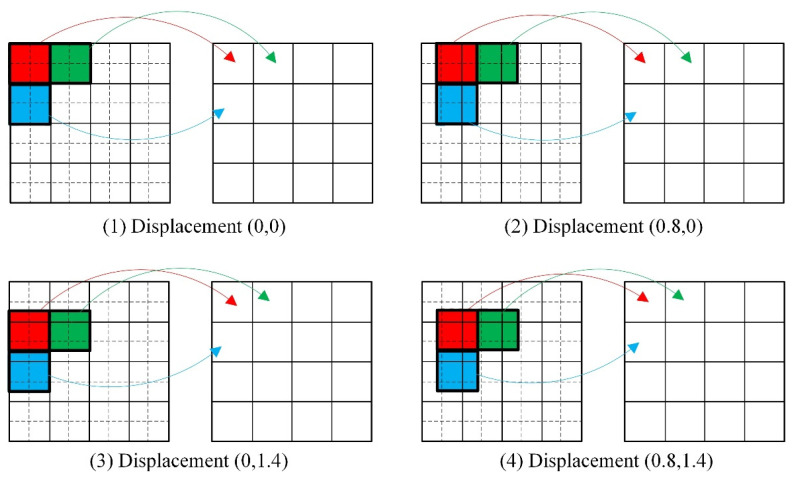
Schematic illustration of the relationship between the HR projection image and the actual LR projection image.

Using the displacement values (0, 0), (0.8, 0), (0, 1.4), and (0.8, 1.4) as an example (Figure 4), the value of an LR pixel value is defined as the sum of the HR pixel values within its corresponding region (outlined by the bold solid-line box). Compared to the ideal case in Figure 3, the coverage of these regions has changed. Consequently, during the summation, the contribution of each HR pixel $\left(x^{\prime },y^{\prime }\right)$ within a region must be weighted by a factor between 0 and 1, which depends on the specific displacement. This implies that a single HR pixel can contribute to multiple LR pixels, creating overlapping areas between the region blocks corresponding to different LR pixels. Based on this model, Equation (2) needs to be reformulated as:

(6) $$P_{L}(x,y)=\sum_{\left(x^{\prime },y^{\prime }\right)\in S_{R}^{*}}w(x^{\prime },y^{\prime })P_{H}^{\prime }(x^{\prime },y^{\prime })$$

**Figure 5:**
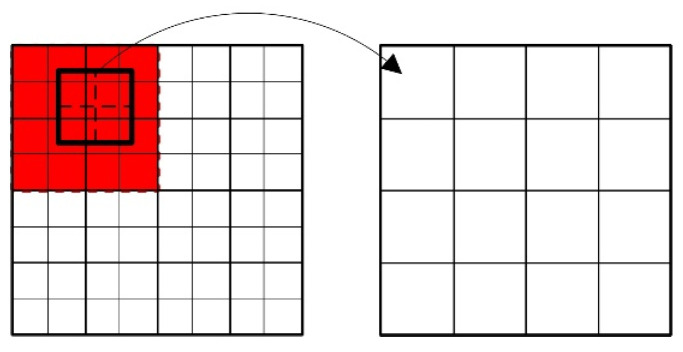
Schematic illustration of the relationship between the HR pixels and the actual LR pixels.

Here, $P_{L}(x,y)$ represents the pixel value at position (x,y) in the LR projection image, while $P_{H}^{\prime }(x^{\prime },y^{\prime })$ denotes the pixel value at position $\left(x^{\prime },y^{\prime }\right)$ in the HR projection image. The summation $\left(x^{\prime },y^{\prime }\right)$ is performed over the corresponding region block $S_{R}^{*}$. $S_{R}^{*}$ represents the new region block, which has a size of (*R* + 2) × (*R* + 2), and $w(x^{\prime },y^{\prime })$ refers to the “contribution” of each point within $S_{R}^{*}$ to the LR projection image pixel, i.e., the weight coefficient. For the LR pixel shown in Figure 5, $S_{R}^{*}$ is represented by the red region.

To express Equation (6) in vector form:

(7) $$p_{L}=WP_{H}^{\prime }$$

here, *p_L_* represents a pixel in the LR projection image; $P_{H}^{\prime }={\left[p_{H_{1}}^{\prime },p_{H_{2}}^{\prime },\cdots ,p_{H_{{\left(R+2\right)}^{2}}}^{\prime }\right]}^{T}$ is a column vector of length (*R* + 2)^2^ × 1, represents the pixels within the region block $S_{R}^{*}$ in the HR projection image; and $W=\left[w_{1},w_{2},\cdots ,w_{{\left(R+2\right)}^{2}}\right]$ is a row vector of length 1 × (*R* + 2)^2^, representing the weight coefficients.

**Figure 6:**
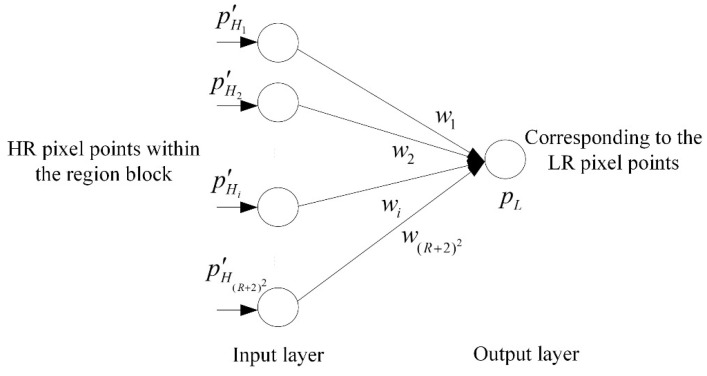
Schematic diagram of the neural network model, where the input is the HR projection data, and the output is the LR projection data.

Equation (7) defines the computational relationship between LR and HR pixel values, with the key being the need to know the weight coefficients. A fixed systematic translation deviation under the LR detector’s known hardware conditions implies that the weighting coefficients are constant. In principle, if both LR and HR pixel values were known, the weight coefficients **W** could be obtained by solving a system of linear equations. In practice, however, deviations in the detector data often result in an ill-conditioned or inconsistent system of equations, rendering a direct solution infeasible. To overcome this, a neural network-based algorithm can be employed to seek an optimal solution. In a neural network, the computation of a neuron corresponds to matrix multiplication, and the product is then passed through an activation function to obtain the output. When the activation function is an identity transformation, the output of the neuron is simply the result of the matrix multiplication, as shown in Figure 6.

For the matrix multiplication shown in Equation (7), when sufficient sample pairs of $p_{L}$ and $P_{H}^{\prime }$ are available, the weight coefficients **W** can be determined by training a neural network. Specifically, a training sample is constructed by using the pixels within a region block $S_{R}^{*}$ of the HR projection image as the neural input, and the corresponding pixel in the LR projection image as the target output. Following this rule, a unique region block can be identified in the HR image for every LR pixel, collectively forming the dataset for training a perceptron. To ensure the model’s generalizability, the design of a comprehensive dataset is detailed in Section 2.4. The complete procedure is outlined in Algorithm 2. Random weight initialization is crucial to break the symmetry of network parameters, preventing them from starting identically. After the network training is completed, the obtained weight coefficients form a 1 × 16 row vector. To map these coefficients to the corresponding pixel positions within the HR image region block $S_{R}^{*}$, they are restored into a two-dimensional matrix form:

(8) $$W=\left[\begin{array}{cccc} w_{1} & w_{2} & w_{3} & w_{4}\\ w_{5} & w_{6} & w_{7} & w_{8}\\ w_{9} & w_{10} & w_{11} & w_{12}\\ w_{13} & w_{14} & w_{15} & w_{16} \end{array}\right]$$

**Algorithm 2.** HR and LR Relationship Acquisition Process**Input**: Training set $T=\left\{\left(P_{H1}^{\prime },p_{L1}\right),\left(P_{H2}^{\prime },p_{L2}\right),\cdots ,\left(P_{HN}^{\prime },p_{LN}\right)\right\}$, where $p_{Li}\in R$ is the value of the LR pixel, $P_{Hi}^{\prime }\in R^{{\left(R+2\right)}^{2}\times 1}$ is the corresponding pixel values within the region block of the HR projection image, i=1,2,…,N, and η is the learning rate. **Output**: The predicted value of the LR pixel ${\tilde{p}}_{Li}\in R$. (1) Randomly select the initial value **W**^(0)^. (2) Select the sample $\left(P_{Hi}^{\prime },p_{Li}\right)$ from the training set. (3) Calculate the output of the perceptron and the mean squared error.                   ${\tilde{p}}_{Li}=WP_{Hi}^{\prime }$                  $Error=\frac{1}{2}\left\|p_{Li}-{\tilde{p}}_{Li}\right\|$ (4) Update the weight coefficients using the SGD method.                $W\leftarrow W-\eta \frac{\partial Error}{\partial W}$ (5) Repeat steps (2) to (4) until the stopping criteria are met (such as reaching the maximum number of iterations or error convergence).

Since each elements of **W** is functionally dependent on the displacement offset (Δx,Δy) between the HR and LR projection images, it can be expressed in terms of this offset. Taking the translation method described in Figure 5 as an example (where the horizontal translation is greater than one pixel and the vertical translation is less than one pixel), the pixel value of an LR projection image is obtained by summing the corresponding 4 pixel values from the HR projection image. These 4 HR pixels are themselves obtained via interpolation within a 4 × 4 region block in the original HR image. Crucially, as the displacement offset varies, the contribution of each pixel within this region block to the final LR pixel value changes accordingly. Let the corresponding region block in **W** be represented by the relationship matrix block **SW**. The elements of **SW** can be expressed as:

(9) $$SW=\left[\begin{array}{cccc} 0 & \left(2-\Delta x)(1-\Delta y\right) & \left(1-\Delta y\right) & \left(\Delta x-1)(1-\Delta y\right)\\ 0 & \left(2-\Delta x\right) & 1 & \left(\Delta x-1\right)\\ 0 & (2-\Delta x)\Delta y & \Delta y & (\Delta x-1)\Delta y\\ 0 & 0 & 0 & 0 \end{array}\right]$$

The weight coefficients **W** are computed by the neural network, while **SW** represents the contribution of each HR pixel value to the LR pixel value during the interpolation process. The two are numerically equal, i.e., **W = SW**.

(10) $$\left[\begin{array}{cccc} w_{1} & w_{2} & w_{3} & w_{4}\\ w_{5} & w_{6} & w_{7} & w_{8}\\ w_{9} & w_{10} & w_{11} & w_{12}\\ w_{13} & w_{14} & w_{15} & w_{16} \end{array}\right]=\left[\begin{array}{cccc} 0 & \left(2-\Delta x)(1-\Delta y\right) & \left(1-\Delta y\right) & \left(\Delta x-1)(1-\Delta y\right)\\ 0 & \left(2-\Delta x\right) & 1 & \left(\Delta x-1\right)\\ 0 & (2-\Delta x)\Delta y & \Delta y & (\Delta x-1)\Delta y\\ 0 & 0 & 0 & 0 \end{array}\right]$$

Therefore, the formula for calculating the offset can be obtained as:

(11) $$\begin{array}{l} \Delta x=\frac{\left(2-w_{6})+(1+w_{8})+(2-(w_{2}+w_{10}))+(1+w_{4}+w_{12}\right)}{4}\\ \Delta y=\frac{\left(1-w_{3})+w_{11}+(w_{10}+w_{12})+(1-(w_{2}+w_{4})\right)}{4} \end{array}$$

In the general case, **W** contains at most 9 non-zero elements, corresponding to the contributions of HR pixels to a given LR pixel value. Once **W** is determined, these 9 non-zero elements and their corresponding weight coefficients U can be extracted, such that:

(12) $$\begin{array}{l} U=\left[\begin{array}{ccc} u_{1} & u_{2} & u_{3}\\ u_{4} & u_{5} & u_{6}\\ u_{7} & u_{8} & u_{9} \end{array}\right]\\ \;\;\;\;=\left[\begin{array}{ccc} \left(q-\Delta x)(p-\Delta y\right) & \left(p-\Delta y\right) & \left(\Delta x-q+1)(p-\Delta y\right)\\ \left(q-\Delta x\right) & 1 & \left(\Delta x-q+1\right)\\ \left(q-\Delta x)(\Delta y-p+1\right) & \left(\Delta y-p+1\right) & \left(\Delta x-q+1)(\Delta y-p+1\right) \end{array}\right] \end{array}$$

where *p* represents the row index of the first non-zero element in **W**, and q represents the column index of the first non-zero element in **W**. Therefore, a more general formula for calculating the offset (Δx,Δy) can be expressed as:

(13) $$\begin{array}{l} \Delta x=\frac{\left(q-u_{4})+(q-1+u_{6})+(q-(u_{1}+u_{7}))+(q-1+u_{3}+u_{9}\right)}{4}\\ \Delta y=\frac{\left(p-u_{2})+(u_{8}+p-1)+(u_{7}+u_{9}+p-1)+(p-(u_{1}+u_{3})\right)}{4} \end{array}$$

To quickly determine which translation relationship corresponds to the obtained **W**, the following strategy is designed: First, set a threshold δ (when the value of an element at a position in **W** is smaller than δ, the element at that position is considered to be zero). When all the elements in the fourth row of **W** are smaller than the threshold δ and all the elements in the fourth column of **W** are smaller than δ, the horizontal and vertical displacement values are both less than one pixel. In this case, both *p* and q are equal to 1. When all the elements in the fourth row of **W** are smaller than the threshold δ and all the elements in the fourth column of **W** are not smaller than the threshold δ, the horizontal displacement is greater than one pixel, and the vertical displacement is smaller than one pixel. In this case, *p* equals 1 and q equals 2. When all the elements in the fourth row of **W** are not smaller than the threshold δ and all the elements in the fourth column of **W** are smaller than the threshold δ, the horizontal displacement is smaller than one pixel, and the vertical displacement is greater than one pixel. In this case, *p* equals 2 and q equals 1. When all the elements in the fourth row of **W** are not smaller than the threshold δ and all the elements in the fourth column of **W** are also not smaller than the threshold δ, both the horizontal and vertical displacements are greater than one pixel. In this case, both *p* and q equal 2.

The general process for calculating the true offset, as described above, is:

(1) Use the neural network to compute the weight coefficients **W**;
(2) Find the non-zero weight coefficients *U* and their corresponding *p* and *q* in **W**;
(3) Use Formula (13) to calculate the true offset (Δx,Δy).

### 2.4. Design of a Randomly Distributed Gamma Point Source

Geometric misalignments due to detector displacement in a multi-detector SPECT system are inherent and remain constant after assembly. To calibrate these translational offsets for subsequent correction within the SR reconstruction algorithm, we designed a dedicated calibration model using randomly distributed gamma point sources. This approach intentionally bypasses tissue attenuation effects, allowing the calibration to focus solely on establishing the precise correspondence between HR and LR projections. This model requires high-activity sources to ensure sufficiently clear point projections (i.e., minimizing noise), A corresponding projection dataset is obtained by imaging this setup. To maximize sample diversity, the model incorporates a variety of radioactive point sources with different intensities and spatial extents, randomly placed throughout the field of view. Furthermore, to facilitate accurate identification of the displacement relationship between their HR and LR projections, the pixel size of each simulated point source is set to be no smaller than 2 × 2 × 2 voxels.

**Figure 7:**
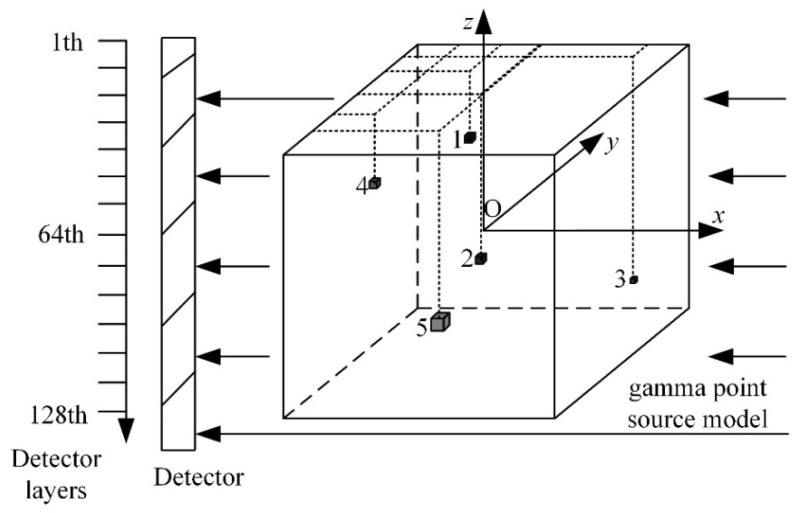
Illustration of the 3D point source model.

The specific parameters of the gamma point source model used in this study are as follows: a 128 × 128 × 128 voxel matrix containing five distinct point sources. It is crucial to note that this is merely an illustrative example. In practical LR detector calibration, once this randomly distributed gamma point source model is utilized, the derived detector geometric error for the parallel-beam LR multi-detector SPECT system becomes universally applicable; it serves to correct the SR reconstruction tasks for any arbitrary object under examination. The coordinates and characteristics of each point source are: The first point source is located at coordinates (35:37, 44:46, 49:51), with a size of 3 × 3 × 3 pixels and an intensity of 1. The second point source is located at coordinates (63:65, 64:66, 79:81), with a size of 3 × 3 × 3 pixels and an intensity of 0.8. The third point source is located at coordinates (15:16, 110:111, 99:100), with a size of 2 × 2 × 2 pixels and an intensity of 0.7. The fourth point source is located at coordinates (79:81, 23:25, 34:36), with a size of 3 × 3 × 3 pixels and an intensity of 0.5. The fifth point source is located at coordinates (96:99, 64:67, 79:82), with a size of 4 × 4 × 4 pixels and an intensity of 0.2. The three-dimensional point source model is illustrated in Figure 7.

## 3. Results

### 3.1. Dataset and Training Details

**Figure 8:**
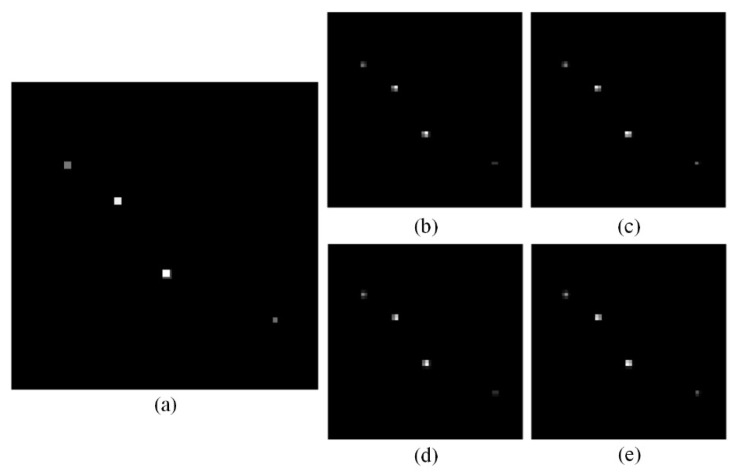
Example of simulated point source projection images. (**a**) HR projection image; (**b**) LR projection image with displacement (0, 0); (**c**) LR projection image with displacement (0.8, 0); (**d**) LR projection image with displacement (0, 1.4); (**e**) LR projection image with displacement (0.8, 1.4).

For the randomly distributed gamma point source model described in Section 2.4, HR and LR projection images at each sampling angle were simulated. This simulation was based on the parallel-beam SPECT imaging geometry, neglecting photon attenuation, and performed using a system matrix and forward projection method. To acquire more projection information, 128 sampling angles are set within the range of 2π. The pixel size of the HR projection image is 128 × 128, while that of the LR projection image is 64 × 64, meaning *R* = 2. Thus, for each sampling angle, 4 LR projection images at different positions need to be obtained. The translation relationship between HR and LR projection images is shown in Figure 4, and the reconstructed image has a pixel size of 128 × 128 × 128. Taking the projection image at the first sampling angle as an example, it is shown in Figure 8.

In addition, in actual data acquisition, the projection data collected by the detector is often accompanied by Poisson noise. This chapter also designs a noisy projection dataset to verify the proposed relationship-finding algorithm and the stability of the reconstruction results. The noise model can be expressed as [28]:

(14) $$\tilde{p}(x,y)\sim \mathrm{Poisson}(kp(x,y))$$

(15) $$\hat{p}(x,y)=\tilde{p}(x,y)/k$$

where p(x,y) represents the noise-free projection data, $\tilde{p}(x,y)$ represents the noisy projection data, $\hat{p}(x,y)$ represents the projection data with noise, accounting for count scaling. The Poisson process is set with equal mean and variance, both denoted as kp(x,y). Here, *k* is set to 1/5 to simulate the intensity of Poisson noise, contaminating the HR projection images of the point source model. The acquisition of LR projections is based on the image degradation model given in Equation (3), where the involved geometric offsets are determined according to Figure 3 and Figure 4. To acquire more training data, 1024 sampling angles are set within the range of π.

Following the procedure outlined in Algorithm 2, the weight coefficient **W** is trained using the HR projection pixels as input and the corresponding LR projection pixels as the target. Subsequently, the true displacement (Δx,Δy) is calculated based on Equations (8)–(13). The projection datasets under different levels of noise contamination were validated separately. The training process was implemented in MATLAB R2021b. Under different offset conditions, training sets are generated by performing matched sampling between HR and LR projections based on the HR-LR relationship described in Figure 5. Samples with LR values of zero should be discarded, and parameters are iteratively optimized using the Stochastic Gradient Descent (SGD) algorithm. When the convergence condition is met, the “contribution” of each point within the region block to the LR pixel, i.e., the weight coefficients W, can be obtained. For the noiseless projection dataset, the learning rate was set to 0.15, the threshold δ was set to 0.001, and the neural network was trained for a total of 200 iterations. For the noise projection dataset, the learning rate was set to 0.05, the threshold δ was set to 0.1, and the neural network was trained for a total of 300 iterations. Once the displacement offset is determined, the SR method from Section 2.2 can be applied to obtain the SR projection image for each sampling angle, ultimately reconstructing the SR image.

### 3.2. Benchmarking

The performance of the proposed displacement estimation method is evaluated based on two criteria. First, its accuracy in determining the offset between HR and LR projections. Second, when applied within the SR SPECT algorithm, its ability to produce SR projection images whose resolution matches that of HR projections corrected with the true displacement offset. These SR projections are then used for final volume reconstruction. In addition, an additional Shepp–Logan model (128 × 128 × 128 pixels) was used. Based on the parallel-beam SPECT imaging principle, HR and LR projection images of the Shepp–Logan model—sized at 128 × 128 and 64 × 64 pixels respectively—are simulated. This is achieved via the forward projection method across 128 angles over a 2π range, using the attenuation map and system matrix. Subsequently, noisy projection data consistent with the random point source training set are generated using Equation (14) to maintain consistent sampling conditions. The Shepp–Logan model is then SR reconstructed using the computed corrected displacement offset, the actual displacement offset, and the uncorrected displacement offset to further evaluate the performance of the proposed method.

### 3.3. Quantitative Assessment

In image processing evaluation standards, Peak Signal-to-Noise Ratio (PSNR) is widely used to evaluate prediction accuracy and is an objective standard for assessing image quality.

(16) $$PSNR=10\log_{10}\frac{\max{\left(u_{n},u_{original_{n}}\right)}^{2}}{\frac{1}{N}\sum_{n=1}^{N}{\left(u_{n}-u_{original_{n}}\right)}^{2}}$$

where $u_{n}$ and $u_{original_{n}}$ are the pixel values of the reconstructed image and the reference image, respectively, and N is the total number of pixels in the image. A larger PSNR means better image quality.

The Structural Similarity Index Measure (SSIM) is an index used to assess the similarity between two images. It is calculated as follows:

(17) $$SSIM=\frac{2{\bar{u}}_{original}\bar{u}(\sigma_{u_{original}u}+c_{2})}{\left({\bar{u}}_{original}^{2}+{\bar{u}}^{2}+c_{1})(\sigma^{2}+\sigma_{original}^{2}+c_{2}\right)}$$

where ${\bar{u}}_{original}$ and $\sigma_{u_{original}}$ are the mean value and variance of the reference image, respectively. $\bar{u}$ and σ are the mean value and variance of the reconstructed image, respectively. $\sigma_{u_{original}u}$ is the covariance of the two images. $c_{1}$ and $c_{2}$ are constants. The closer the SSIM value is to 1, the higher the similarity between the images.

In addition, to evaluate the imaging quality, a region of interest (ROI) was often selected in the image to calculate the contrast recovery coefficient (CRC):

(18) $$CRC=\frac{\left(\frac{\bar{ROI}}{\bar{B}}-1\right)}{\left(\frac{{\bar{ROI}}_{original}}{{\bar{B}}_{original}}-1\right)}$$

where $\bar{ROI}$ is the mean value of the ROI in the reconstructed image and $\bar{B}$ is the mean value of the background in the reconstructed image. ${\bar{ROI}}_{original}$ is the mean value of the ROI in the reference image, and ${\bar{B}}_{original}$ is the mean value of the background in the reference image.

### 3.4. Obtaining the Offset of LR Detector Through Gamma Point Source

For the noise-free random point source projection dataset, the actual computed relationship between HR and LR projection images is shown in Table 1. In the absence of noise, the displacement offset between HR and LR projection images can be calculated using the neural network algorithm, with the deviation between the calculated displacement offset and the theoretical value on the order of 10^−6^. Table 2 shows the displacement deviation between HR and LR projection images obtained using the neural network algorithm under Poisson noise. The results indicate that the deviation between the calculated displacement offset and the theoretical value is on the order of 10^−2^.

### 3.5. SR Reconstruction of a Gamma Point Source Model

**Figure 9:**
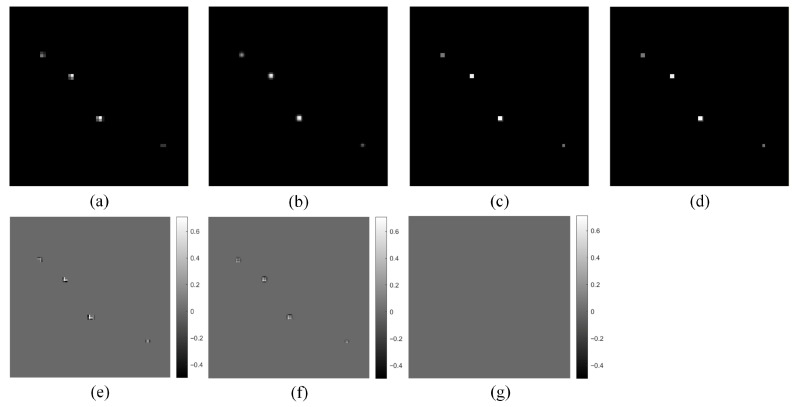
Comparison of SR projection images obtained at a single sampling angle. (**a**) LR projection; (**b**) SR projection obtained using uncorrected offset; (**c**) SR projection obtained using our corrected offset; (**d**) HR projection; (**e**) residual map between LR projection and HR projection; (**f**) residual map between SR projection obtained using uncorrected offset and HR projection; (**g**) residual map between SR projection obtained using our corrected offset and HR projection.

For each sampling angle, the 4 LR projection images were processed using the corrected displacement offset from Table 1, and the corresponding HR projection images for each angle can be obtained using the SR algorithm described in Section 2.2. Taking the first sampling angle’s projection image as an example, the uncorrected HR projection image, obtained by using the uncorrected displacement offset, is compared with the corrected HR projection image, obtained by using the corrected displacement offset, as well as the directly projected HR projection image and the LR projection image, as shown in Figure 9. All images are normalized after optimization of their respective iteration parameters.

The SR projection image obtained using our corrected offset is almost identical to the true HR projection image, while the SR projection image obtained using uncorrected offset, although better than the LR projection image, deviates significantly from the true HR projection image. This discrepancy originates from the simulation process: when a biased offset is used to generate the HR projection, the subsequent downsampling produces a simulated LR image that systematically deviates from the actual LR data. This persistent error impedes convergence during iteration process, ultimately affecting the final result.

**Figure 10:**
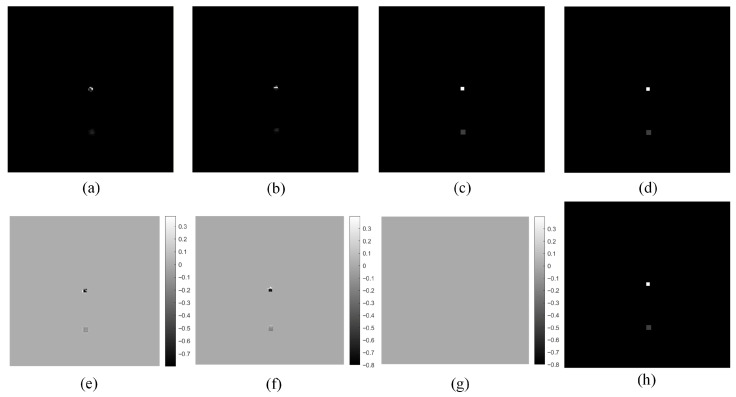
Example of point source model reconstructed images obtained by different SR projections in Figure 9. (**a**) Reconstructed image from LR projection; (**b**) reconstructed image from SR projection obtained using uncorrected offset; (**c**) reconstructed image from SR projection obtained using our corrected offset; (**d**) reconstructed image from HR projection; (**e**) residual map between (**a**,**d**); (**f**) residual map between (**b**,**d**); (**g**) residual map between (**c**,**d**); (**h**) point source model.

Using the MLEM reconstruction algorithm, 3D reconstructed images were obtained from the projection images acquired by different methods. Figure 10 shows the reconstruction results at slice 80, corresponding to the position of the radioactive source. It is observed that both the LR reconstruction result and the uncorrected projection reconstruction result fail to fully restore the point source information. However, the reconstruction image obtained by using the corrected projection yields results similar to the HR reconstruction image, with the smallest residual between the corrected reconstruction image and the HR reconstruction image.

**Figure 11:**
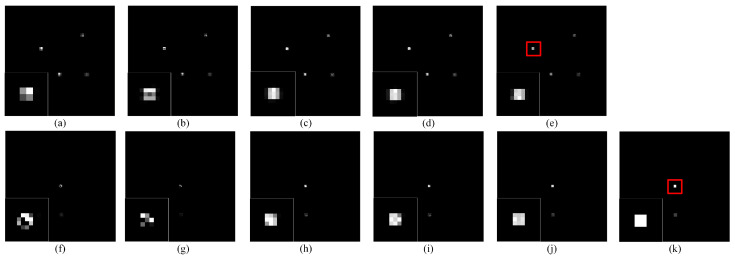
Projection and reconstructed images of the point source model obtained through SR methods with different displacement offsets under Poisson noise. (**a**) LR projection; (**b**) SR projection obtained using uncorrected offset; (**c**) SR projection obtained using our corrected offset; (**d**) SR projection obtained using actual corrected offset; (**e**) HR projection; (**f**) reconstructed image from LR projection; (**g**) reconstructed image from SR projection obtained using uncorrected offset; (**h**) reconstructed image from SR projection obtained using our corrected offset; (**i**) reconstructed image from SR projection obtained using actual corrected offset; (**j**) reconstructed image from HR projection; (**k**) point source model.

The results for point source data with Poisson noise are presented in Figure 11. The projection image is selected from the 39th sampling angle for display, and the reconstructed image is displayed from the 80th slice. In each subfigure, the region indicated by the red box is magnified in the lower-left corner for detailed inspection. The zoomed-in data in the projection images shows that, under Poisson noise, the use of uncorrected displacement offset leads to amplified displacement errors, causing pixel discontinuities in the projection image. The SR reconstruction image obtained using the displacement offset derived from the proposed method shows better results than the uncorrected reconstruction image, and its quality is close to the SR reconstruction image obtained using the actual offset. Both can effectively capture the contour information of the point source.

### 3.6. SR Reconstruction of a Shepp–Logan Model

**Figure 12:**
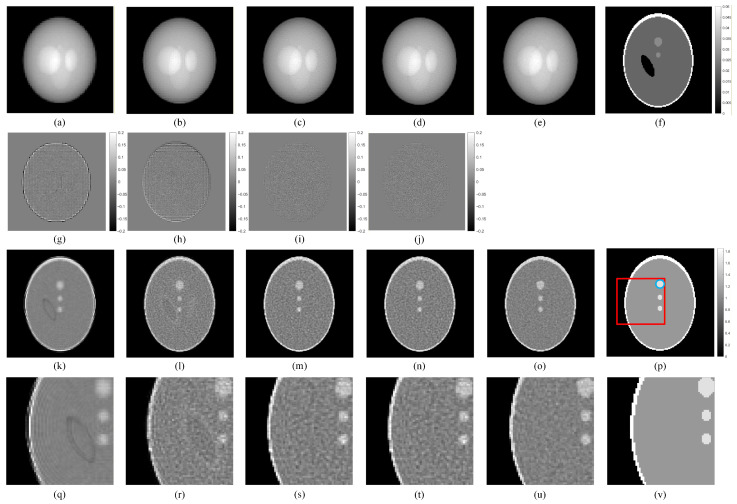
Projection and reconstruction images of the Shepp–Logan model with different displacement offsets under Poisson noise. (**a**) LR projection; (**b**) SR projection obtained using uncorrected offset; (**c**) SR projection obtained using our corrected offset; (**d**) SR projection obtained using actual corrected offset; (**e**) HR projection; (**f**) attenuation map; (**g**) residual map between (**a**,**e**); (**h**) residual map between (**b**,**e**); (**i**) residual map between (**c**,**e**); (**j**) residual map between (**d**,**e**); (**k**) reconstructed image from LR projection; (**l**) reconstructed image from SR projection obtained using uncorrected offset; (**m**) reconstructed image from SR projection obtained using our corrected offset; (**n**) reconstructed image from SR projection obtained using actual corrected offset; (**o**) reconstructed image from HR projection; (**p**) Shepp–Logan model. (**q**) region in (**k**) that matches the position of the magnified red-boxed region in (**p**); (**r**) region in (**l**) that matches the position of the magnified red-boxed region in (**p**); (**s**) region in (**m**) that matches the position of the magnified red-boxed region in (**p**); (**t**) region in (**n**) that matches the position of the magnified red-boxed region in (**p**); (**u**) region in (**o**) that matches the position of the magnified red-boxed region in (**p**); (**v**) magnified red-boxed region from (**p**).

The results for the Shepp–Logan model under Poisson noise are shown in Figure 12. The projection image is selected from the first sampling angle, and the reconstruction image is displayed at the 50th slice. The ordered subset expectation maximization (OSEM) reconstruction algorithm is used, with image reconstruction performed over 8 iterations, and 16 subsets per iteration. From the residual maps between the projection images obtained by different methods and the HR projection images, it can be observed that the projection images obtained using the uncorrected displacement offset have slightly better quality than the LR projection images, but still lower than the projection images obtained using the corrected displacement offset. The projection images obtained using the uncorrected displacement offset contain more pixelization differences, which is similar to the results from the point source model experiment. This is particularly evident in the boundary areas of the images.

**Figure 13:**
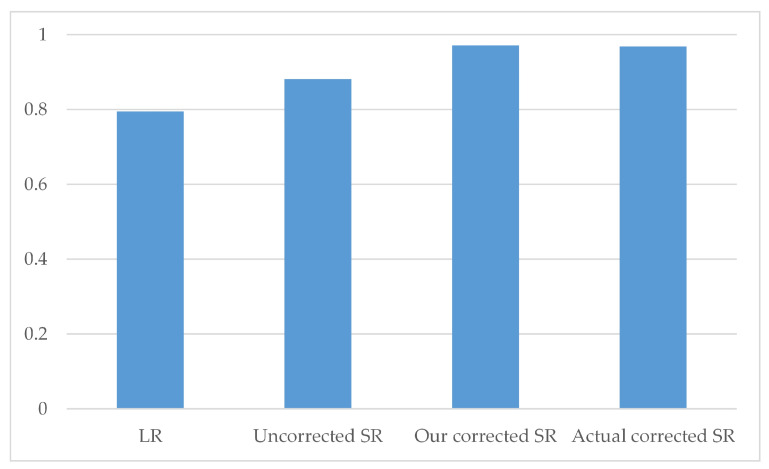
Average CRC metrics for different processing methods (for ROI indicated by blue circle in Figure 12).

For the reconstructed images, the images obtained using our displacement offset are similar to those obtained with the actual displacement offset. The images show no significant artifacts and are of quality comparable to the HR reconstructed images. The uncorrected reconstructed images and LR reconstructed images exhibit relatively poor boundary conditions and fail to effectively perform attenuation correction. By magnifying the data within the red box in the images, the difference can be more clearly observed. Finally, the image quality of different methods is evaluated using PSNR and SSIM, as shown in Table 3. The proposed method achieves evaluation values similar to those of the actual corrected displacement offset, and it performs better than the uncorrected images. Although the LR image has a higher SSIM, its visual quality is the worst, which is attributed to the excessively smooth pixel values in the LR image. We selected the region marked by the blue circle in Figure 12p as the ROI. As shown in Figure 13, the reconstructed image from the SR projection obtained using our corrected offset achieves a comparable CRC magnitude to that obtained using the actual corrected offset, and both are significantly superior to the reconstructed image from the LR projection and the reconstructed image from the SR projection obtained using the uncorrected offset.

## 4. Discussion

Numerical simulation results demonstrate that the SR images reconstructed using the proposed method, corrected with the estimated displacement offset, achieve quality comparable to those reconstructed using the actual displacement offset. Furthermore, these corrected images closely approximate the quality of genuine HR projection reconstruction images. This confirms that the proposed neural network method can accurately determine the displacement bias between HR and LR projection images in the three-dimensional parallel-beam SPECT SR task, resulting in an approximate two-fold improvement in SPECT image resolution. As observed in Figure 12, the displacement bias between HR and LR images has minimal impact on regions with continuous pixel values in the reconstructed images. This phenomenon may be attributed to two factors: firstly, Poisson noise could partially mask the error; alternatively, the smooth and continuous nature of the image might allow the displacement bias to propagate, shifting its visible effects toward boundary areas or regions with significant pixel value differences, such as the edges and attenuation regions shown in Figure 12.

**Figure 14:**
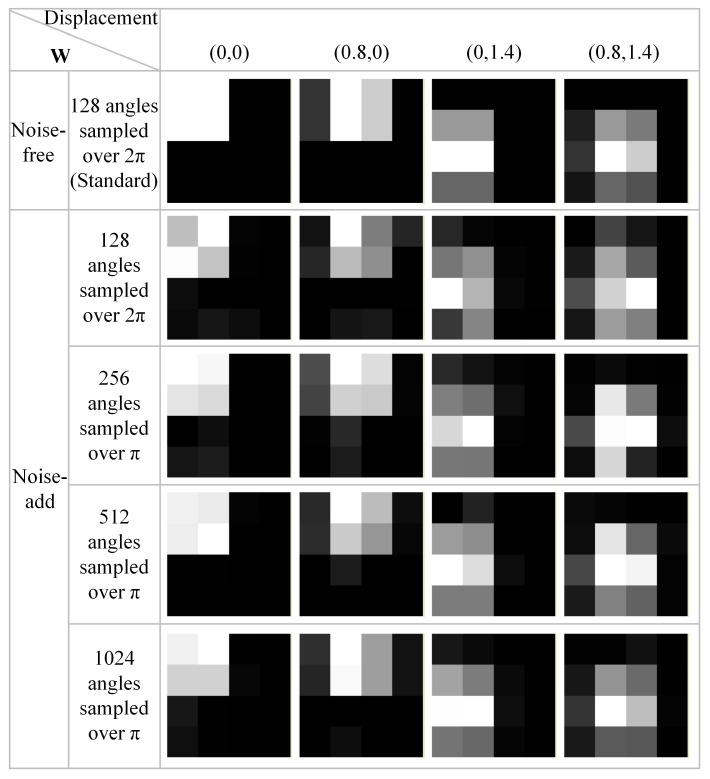
Visualization of the calculated weight coefficients W under different training data volumes.

Our findings indicate that point source datasets with Poisson noise require a larger volume of training data. As the number of sampling angles increases from 128 to 256, 512, and finally 1024, the relationship matrix obtained through training gradually approaches the weight coefficients **W** from the noise-free dataset. The weight coefficients corresponding to different training datasets are visualized in Figure 14. In the presence of Poisson noise, the threshold value δ in the proposed neural network-based SR method is empirically chosen. For the noise level described in this study, δ is set to 0.1, corresponding to 10% of the HR pixel size, allowing an error tolerance of 0.1. Experimental results also indicate that the error is approximately 10^−2^. The proposed neural network method runs in MATLAB R2021b. Owing to the large number of zero elements in the projection data of the random point source model, the training convergence speed is relatively fast. After 300 iterations, obtaining the relationship matrix for a single displacement position task only about 10 s. In practical applications, acceptable computational accuracy can be achieved by configuring reasonable thresholds and iteration counts—though this necessitates extensive validation in real-world scenarios. Our work has primarily validated the principles of the proposed method. In future studies, parameter selection will depend on the experimental conditions of different systems. Therefore, parameter selection must ultimately be tailored to the specific experimental conditions of each imaging system.

The use of a high-intensity random point source dataset for detector geometric calibration simplifies the consideration of HR-LR projection relationships while also enabling faster training, fewer iterations, and lower computational costs. Once trained, the obtained offset parameters for the LR detectors can be applied to any target within the same system, eliminating the need for retraining on each newly acquired SPECT projection. Furthermore, the high-intensity radioactive source ensures projection datasets are acquired under low Poisson noise levels, thereby enhancing the accuracy of the method. However, this approach introduces a greater radiation burden. In practical applications, the activity of the point source should be adjusted according to specific needs, and increasing the number of random point source models may be considered when necessary. It should be noted that these simulation results are preliminary, aimed at validating the principle of the proposed method under ideal conditions rather than simulating the full SPECT imaging process. Future work will involve more realistic physical models and experimental validation on actual hardware systems.

## 5. Conclusions

In this study, we propose a parallel-beam multi-LR-detector SPECT concept. By arranging multiple LR detectors with relative sub-pixel shifts within the sampling space beforehand, this design eliminates the need for additional acquisition time and large-scale mechanical motion, thereby improving overall efficiency. For such a system, we present a super-resolution (SR) reconstruction method to synthesize high-resolution (HR) SPECT images from the LR projections acquired by the four shifted detectors. To correct for geometric errors among these detectors, we designed a randomly distributed gamma point source to generate training data. Subsequently, a neural network-based algorithm was introduced to estimate the geometric errors, which optimizes the SR reconstruction process. Numerical simulations confirm that this approach can double the image resolution. It should be noted that these simulation results serve as a preliminary validation of the SR reconstruction principle under the proposed scheme, and further testing with actual equipment is warranted.

## Figures and Tables

**Table 1:** Computational relationship between HR and LR projection images (noise free).

LR Detector	Computed Coefficient W	Corrected Offset	Actual Offset	Uncorrected Offset
Displacement offset (0, 0)	$\left[\begin{array}{cccc} 1.0000 & 1.0000 & 0 & 0\\ 1.0000 & 0.9999 & 0 & 0\\ 0 & 0 & 0 & 0\\ 0 & 0 & 0 & 0 \end{array}\right]$	Δ*x* = 0.000001 Δ*y* = 0.000004	Δ*x* = 0.000000 Δ*y* = 0.000000	Δ*x* = 0.000000 Δ*y* = 0.000000
Displacement offset (0.8, 0)	$\left[\begin{array}{cccc} 0.2000 & 1.0000 & 0.8000 & 0\\ 0.2000 & 1.0000 & 0.8000 & 0\\ 0 & 0 & 0 & 0\\ 0 & 0 & 0 & 0 \end{array}\right]$	Δ*x* = 0.799999 Δ*y* = 0.000004	Δ*x* = 0.800000 Δ*y* = 0.000000	Δ*x* = 1.000000 Δ*y* = 0.000000
Displacement offset (0, 1.4)	$\left[\begin{array}{cccc} 0 & 0 & 0 & 0\\ 0.6000 & 0.6000 & 0 & 0\\ 1.0000 & 1.0000 & 0 & 0\\ 0.4000 & 0.4000 & 0 & 0 \end{array}\right]$	Δ*x* = 0.000001 Δ*y* = 1.400000	Δ*x* = 0.000000 Δ*y* = 1.400000	Δ*x* = 0.000000 Δ*y* = 1.000000
Displacement offset (0.8, 1.4)	$\left[\begin{array}{cccc} 0 & 0 & 0 & 0\\ 0.1200 & 0.6000 & 0.4800 & 0\\ 0.2000 & 1.0000 & 0.8000 & 0\\ 0.0800 & 0.4000 & 0.3200 & 0 \end{array}\right]$	Δ*x* = 0.800000 Δ*y* = 1.400000	Δ*x* = 0.800000 Δ*y* = 1.400000	Δ*x* = 1.000000 Δ*y* = 1.000000

**Table 2:** Computational relationship between HR and LR projection images (noise added).

LR Detector	Computed Coefficient W	Corrected Offset	Actual Offset	Uncorrected Offset
Displacement offset (0, 0)	$\left[\begin{array}{cccc} 0.9595 & 1.0584 & 0.0153 & 0.0064\\ 0.9829 & 0.7950 & 0.0521 & 0.0020\\ 0.0037 & 0.0188 & 0.0155 & 0.0012\\ 0.0510 & 0.0090 & 0.0031 & 0.0010 \end{array}\right]$	Δ*x* = 0.034218 Δ*y* = 0.001218	Δ*x* = 0.000000 Δ*y* = 0.000000	Δ*x* = 0.000000 Δ*y* = 0.000000
Displacement offset (0.8, 0)	$\left[\begin{array}{cccc} 0.2026 & 1.0567 & 0.8239 & 0.0300\\ 0.2026 & 0.9261 & 0.6932 & 0.0236\\ 0.0181 & 0.0214 & 0.1064 & 0.0085\\ 0.0000 & 0.0005 & 0.0014 & 0.0021 \end{array}\right]$	Δ*x =* 0.800056 Δ*y* = 0.015662	Δ*x* = 0.800000 Δ*y* = 0.000000	Δ*x* = 1.000000 Δ*y* = 0.000000
Displacement offset (0, 1.4)	$\left[\begin{array}{cccc} 0.0983 & 0.0424 & 0.0257 & 0.0029\\ 0.6129 & 0.4896 & 0.0699 & 0.0050\\ 0.9209 & 0.9660 & 0.0640 & 0.0041\\ 0.4237 & 0.3634 & 0.0225 & 0.0054 \end{array}\right]$	Δ*x* = 0.049726 Δ*y* = 1.409296	Δ*x* = 0.000000 Δ*y* = 1.400000	Δ*x* = 0.000000 Δ*y* = 1.000000
Displacement offset (0.8, 1.4)	$\left[\begin{array}{cccc} 0.0110 & 0.0207 & 0.0879 & 0.0059\\ 0.0908 & 0.6108 & 0.4340 & 0.0090\\ 0.2158 & 0.9863 & 0.7613 & 0.0253\\ 0.1175 & 0.3334 & 0.3546 & 0.0103 \end{array}\right]$	Δ*x* = 0.781458 Δ*y* = 1.417465	Δ*x* = 0.800000 Δ*y* = 1.400000	Δ*x* = 1.000000 Δ*y* = 1.000000

**Table 3:** Comparison of PSNR and SSIM for different results.

		LR	Uncorrected SR	Our Corrected SR	Actual Corrected SR
Projection image	PSNR	33.11	35.46	39.62	39.64
	SSIM	0.9410	0.9334	0.9495	0.9493
Reconstructed image	PSNR	24.58	27.26	28.29	28.39
	SSIM	0.7454	0.7043	0.7201	0.7186

## Data Availability

The data presented in this study are available on request from the corresponding author due to ongoing and planned future studies.

## Abbreviations

The following abbreviations are used in this manuscript:

CRC	Contrast recovery coefficient
DL	Deep learning
FOV	Field of view
HR	High-resolution
KEM	Kernelized expectation maximization
LR	Low-resolution
MLEM	Maximum Likelihood Expectation Maximization
ROI	Region of interest
TV	Total variation
SPECT	Single photon emission computed tomography
SR	Super-resolution
